# Supplementary material for: Comparative multiomics analysis of cell physiological state after culture in a basket bioreactor
Source: Sci Rep. 2022 Nov 23;12:20161. doi: 10.1038/s41598-022-24687-4 (PMC9686226; doi:10.1038/s41598-022-24687-4)
Supplement: Supplementary file 1 — Supplementary Information 1. [file 41598_2022_24687_MOESM1_ESM.zip › raw data/Transcriptomic raw data/6.Enrichment/1.GO/TE1vsTC1/GSEA/TC1/ORGANOPHOSPHATE_CATABOLIC_PROCESS/ORGANOPHOSPHATE_CATABOLIC_PROCESS(GO_0046434).html]

Details for gene set ORGANOPHOSPHATE\_CATABOLIC\_PROCESS(GO:0046434)[GSEA]


|  || Dataset | readcount.sample |
| Phenotype | sample.cls |
| Upregulated in class | TC1 |
| GeneSet | ORGANOPHOSPHATE\_CATABOLIC\_PROCESS(GO:0046434) |
| Enrichment Score (ES) | -0.14641455 |
| Normalized Enrichment Score (NES) | -0.90362936 |
| Nominal p-value | 0.52134144 |
| FDR q-value | 0.94824547 |
| FWER p-Value | 1.0 |
Table: GSEA Results Summary

  

Fig 1: Enrichment plot: ORGANOPHOSPHATE\_CATABOLIC\_PROCESS(GO:0046434)
  
Profile of the Running ES Score & Positions of GeneSet Members on the Rank Ordered List

  

| PROBE | DESCRIPTION   (from dataset) | GENE SYMBOL | GENE\_TITLE | RANK IN GENE LIST | RANK METRIC SCORE | RUNNING ES | CORE ENRICHMENT || 1 | 103232107 | LOC103232107 |  |  | 235 | Infinity | -0.0101 | No |
| 2 | 119623417 | LOC119623417 |  |  | 1788 | 2.248 | 0.0171 | No |
| 3 | 103217648 | TMEM237 |  |  | 4110 | 1.514 | -0.0217 | No |
| 4 | 103242214 | ENO3 |  |  | 4874 | 1.414 | 0.0049 | No |
| 5 | 103241822 | GPD1L |  |  | 5432 | 1.357 | 0.0383 | No |
| 6 | 103238217 | PFKM |  |  | 6902 | 1.229 | 0.0254 | No |
| 7 | 103240047 | LOC103240047 |  |  | 7696 | 1.166 | 0.0400 | No |
| 8 | 103246100 | PLA2G4B |  |  | 11866 | 0.902 | -0.1077 | Yes |
| 9 | 103230811 | EDARADD |  |  | 12094 | 0.889 | -0.0797 | Yes |
| 10 | 103232238 | PGK1 |  |  | 12477 | 0.864 | -0.0597 | Yes |
| 11 | 103236549 | LOC103236549 |  |  | 12544 | 0.860 | -0.0257 | Yes |
| 12 | 103243434 | LOC103243434 |  |  | 12790 | 0.845 | -0.0004 | Yes |
| 13 | 103237780 | LOC103237780 |  |  | 13638 | 0.793 | -0.0042 | Yes |
| 14 | 103230584 | LOC103230584 |  |  | 14738 | 0.725 | -0.0222 | Yes |
| 15 | 119623700 | LOC119623700 |  |  | 14837 | 0.718 | 0.0043 | Yes |
| 16 | 103216571 | ENO4 |  |  | 14869 | 0.716 | 0.0336 | Yes |
| 17 | 103217002 | LOC103217002 |  |  | 15680 | 0.663 | 0.0259 | Yes |
| 18 | 103236321 | LOC103236321 |  |  | 15792 | 0.656 | 0.0491 | Yes |
| 19 | 103246072 | PKM |  |  | 16089 | 0.635 | 0.0631 | Yes |
| 20 | 103219799 | PFKL |  |  | 16212 | 0.628 | 0.0846 | Yes |
| 21 | 103234470 | GPI |  |  | 16493 | 0.605 | 0.0981 | Yes |
| 22 | 103238296 | GPD1 |  |  | 17505 | 0.532 | 0.0757 | Yes |
| 23 | 103242471 | NT5M |  |  | 17742 | 0.511 | 0.0871 | Yes |
| 24 | 119621354 | LOC119621354 |  |  | 18273 | 0.463 | 0.0833 | Yes |
| 25 | 103230418 | PLA2G4A |  |  | 18290 | 0.461 | 0.1024 | Yes |
| 26 | 119618100 | ALDOA |  |  | 18765 | 0.414 | 0.0989 | Yes |
| 27 | 103240043 | NT5E |  |  | 19263 | 0.364 | 0.0924 | Yes |
| 28 | 103218486 | ENO2 |  |  | 19420 | 0.349 | 0.1004 | Yes |
| 29 | 103235528 | PLA2G4C |  |  | 19975 | 0.276 | 0.0875 | Yes |
| 30 | 103243056 | NT5C |  |  | 20232 | 0.236 | 0.0862 | Yes |
| 31 | 119623898 | LOC119623898 |  |  | 20707 | 0.147 | 0.0713 | Yes |
| 32 | 103242625 | ALDOC |  |  | 20901 | 0.100 | 0.0670 | Yes |
| 33 | 103245774 | PLA2G4F |  |  | 21401 | 0.000 | 0.0447 | Yes |
| 34 | 103245777 | PLA2G4E |  |  | 22010 | 0.000 | 0.0175 | Yes |
| 35 | 103219114 | ALDOB |  |  | 22052 | 0.000 | 0.0156 | Yes |
Table: GSEA details
[plain text format]

  

Fig 2: ORGANOPHOSPHATE\_CATABOLIC\_PROCESS(GO:0046434)
  
Blue-Pink O' Gram in the Space of the Analyzed GeneSet

  

Fig 3: ORGANOPHOSPHATE\_CATABOLIC\_PROCESS(GO:0046434): Random ES distribution
  
Gene set null distribution of ES for
 **ORGANOPHOSPHATE\_CATABOLIC\_PROCESS(GO:0046434)**
